# Combined and selective miR-21 silencing and doxorubicin delivery in cancer cells using tailored DNA nanostructures

**DOI:** 10.1038/s41419-020-03339-3

**Published:** 2021-01-07

**Authors:** Sofia Raniolo, Valeria Unida, Giulia Vindigni, Carmine Stolfi, Federico Iacovelli, Alessandro Desideri, Silvia Biocca

**Affiliations:** 1 Department of Systems Medicine, University of Rome Tor Vergata, Via Montpellier 1, 00133 Rome, Italy; 2 Department of Biology, University of Rome Tor Vergata, Via della Ricerca Scientifica 1, 00133 Rome, Italy

**Keywords:** Nucleic-acid therapeutics, Cancer therapy

## Abstract

MicroRNAs play an important role in tumorigenesis and, among them, miR-21 is found to be aberrantly up-regulated in various tumors. The tumor-associated antigen, folate receptor alpha is a GPI-membrane protein overexpressed in many malignant tumors of epithelial origin, including ovarian and cervical cancers. Covalently bound octahedral DNA nanocages were functionalized with folate molecules and utilized as scaffolds to engineer four sequestering units with a miR-21 complementary sequence for obtaining biocompatible Fol-miR21-NC non-toxic nanostructures, to be able to selectively recognize folate receptor alpha-overexpressing cancer cells and sequester the oncogenic miR-21. qPCR assays showed that Fol-miR21-NCs reduce the miR-21 expression up to 80% in cancer cells in the first 2 days of treatment. Functional assays demonstrated that miR-21 sequestering leads to up-regulation of miR-21 tumor suppressor targets (i.e., PTEN and Pdcd4), reduction in cancer cell migration, reduction in proliferation, and increase in cell death. Fol-miR21-NCs can be efficiently loaded with the chemotherapeutic agent doxorubicin. Co-delivery of anti-miR-21 and doxorubicin showed additive cytotoxic effects on tumor cells, paving the way for their use as selective nucleic acid drugs.

## Introduction

Aberrant expression of microRNAs (miRNAs) has been reported in various tumors indicating that there is a close correlation between miRNAs and human malignancy. MiRNAs are involved in many biological processes, such as cell proliferation and apoptosis, by regulating gene expression at post-transcriptional level^1^. Notably, dysregulation in the expression of different miRNAs contributes to cancer development and progression^2–7^. MiR-21 was found to be consistently up-regulated in clinical samples from cancer patients^8,9^ and, in some cancer cell lines, it represents up to 15–25% of the total cellular miRNA content^10^. Dysregulation of miR-21 expression is related with the proliferation, apoptosis, and migration of cancer cells^9^. The increase in miR-21 levels in cancer cells was found to down-regulate tumor suppressor proteins, PTEN and programmed cell death protein 4 (Pdcd4), and to regulate different apoptotic genes^10^. These observations suggest miR-21 knockdown as a promising anti-cancer therapeutic strategy. However, it is worth mentioning that miR-21 inhibition has been reported to be cytotoxic in several non-cancerous cell types^11^. Thus, to minimize off target side effects and avoid unexpected collateral damage in normal cells, a targeted therapy becomes crucial^12^.

The advent of nanotechnology in biomedicine has led to new opportunities in cancer therapy, offering the possibility to use innovative drug delivery systems^13^. DNA-based nanostructures (DNS) are at the forefront of emerging technologies for drug delivery, gene silencing, and diagnostic imaging as well as for studying molecular recognition and signal transduction processes^14^. Due to the intrinsic biocompatibility, structural flexibility, and stability of the polymer, DNA strands are good candidates for assembling DNS with different geometries and sizes^15^. DNS have been engineered to control switchable open/close mechanisms and functionalized to include cellular recognition signals, such as folate, peptides, or aptamers, for generating nanostructures selectively targeting cancer cells through receptor-mediated mechanisms^16–19^. DNS have been also applied for miRNA detection, for overexpressed oncomiRs knockdown, or for tumor suppressive miRNA delivery^20^. Notably, neither intrinsic toxicity nor immune response have been detected after systemic injection of DNS into mouse models^21^. Our group has been deeply involved in the design, assembly, and structural-dynamical characterization of DNS with a specific geometrical arrangement, namely truncated octahedral DNA nanocages^22,23^, identifying the parameters that can modulate the yield of assembly and their mechanical-dynamical properties^24^. We characterized different types of fully covalently octahedral DNA nanocages and studied the receptor-mediated cell entry and their efficacy in selective drug delivery^25–27^. Among receptors, we focused on the α-isoform of the folate receptor (αFR), a tumor-associated antigen highly expressed in many malignant tumors and largely absent in normal tissues. In general, folate-conjugation has great therapeutic potential for small RNAs and drug delivery, and for selective targeting of DNS to αFR-overexpressing cells^28^.

Doxorubicin (Dox) is one of the most efficacious chemotherapeutic agent used for a wide variety of solid tumors and hematological malignancies^29^. Dox intercalates into DNA double helix causing multiple toxic effects, such as prevention of DNA replication, disruption of topoisomerase-II-mediated DNA repair, and generation of reactive oxygen species^30^. Notably, DNS can be loaded with Dox and used as nanocarriers for drug delivery. In particular, folate-functionalized DNA nanocages (Fol-NCs) loaded with Dox induce a selective toxicity to cancer cells overexpressing the folate receptor^26^.

We recently obtained openable nanostructures that are able to recognize and bind specific oligonucleotide sequences in vitro and in cells, suitable for specifically sequestering intracellular oligonucleotides, such as miRNAs^31^. Here, we propose folate-functionalized NCs harboring DNA sequestering units complementary to miR-21 (Fol-miR21-NCs), to form nanostructures with a specific miR-21 silencing activity. HeLa and IGROV1 cell lines were used as in vitro cervical and ovarian tumor models, since both cell lines overexpress miR-21^32,33^ and present high levels of the αFR^26,34^. We have analyzed time-dependent internalization, intracellular stability, targeting selectivity, and downstream gene regulation of Fol-miR21-NCs, evaluating their therapeutic efficacy following cell migration inhibition and cell death. Finally, Fol-miR21-NCs were loaded with Dox for evaluating the therapeutic efficacy of a combined treatment of miR-21 silencing and Dox release.

## Results

### Models of the unbound/bound states of Fol-miR21-NC

**Fig. 1:**
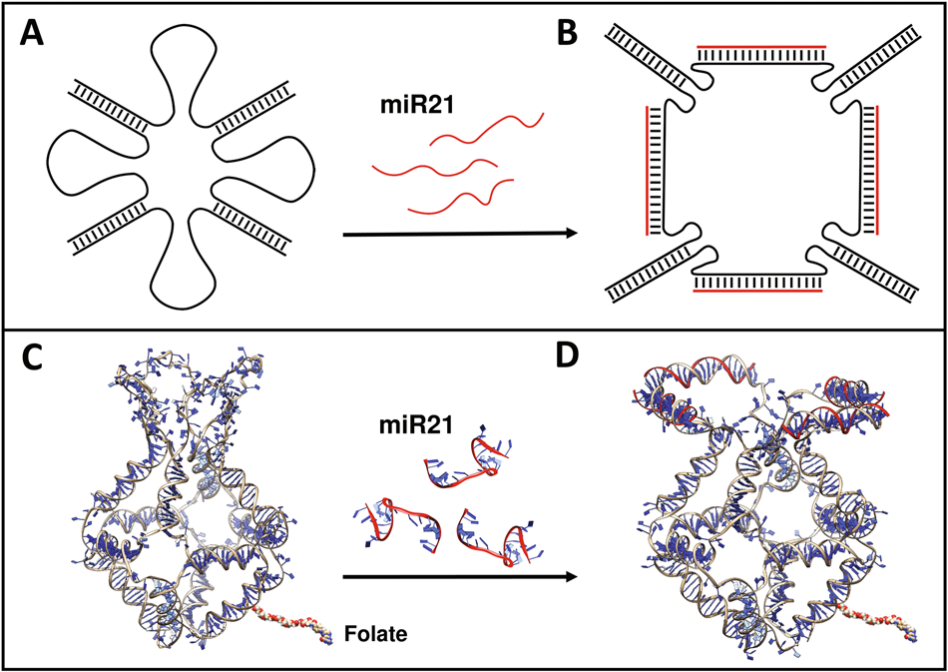
Schematic and atomistic representation of Fol-miR21-NC. **A** Schematic top view of the four sequestering units in the unbound state. **B** Schematic top view highlighting the bound state of Fol-NC upon interaction with miR-21 molecules. **C, D** Three-dimensional representation of the two states, showing the conformational change occurring in the Fol-miR21-NC after binding to miR-21.

The Fol-miR21-NC was designed starting from a recently described H4-DNA cage^31^, where four miRNA sequestering units were introduced in a corner of a truncated octahedral DNA nanocage^22^. The structure, resulting from the assembly of eight oligonucleotides, is characterized by a scaffold consisting of 12 double-stranded B-DNA helices, which form the main edges, connected by short single-stranded five-thymidine linkers, constituting the square truncated faces (Supplementary Fig. 1). The four sequestering units have 21-nucleotide DNA sequence, complementary to mature miR-21-5p, connected to the cage scaffold through two 12-nucleotide linkers made by random TC sequences. Binding of miRNAs induces a conformational change to the Fol-NC, switching it from an unbound (Fig. 1A, C) to a bound conformation (Fig. 1B, D). A 21-nucleotide random (scramble) DNA sequence was used to assemble a non-relevant Fol-scr-NC to be used as negative control. For a selective receptor-mediated cell uptake, a single oligonucleotide has been functionalized with folate, to obtain folate-functionalized DNA nanocages^26,27^.

### Internalization and stability of Fol-NCs in cancer cells

Folate-functionalized nanostructures were assembled as described earlier^22,25^ and functionalized with a biotin molecule (Bio), for their detection through the biotin-streptavidin assays. To evaluate the time-dependent cellular uptake and stability of Fol-NCs, we used αFR-overexpressing HeLa and IGROV1 cells as in vitro tumor models^26,34^. It is worth mentioning that Fol-miR21-NCs and Fol-scr-NCs differ only for the four sequestering sequences. They are fully stable for at least 4 h in 10% FBS and then they slowly start to be degraded being still detectable at 24 h (Supplementary Fig. 2). As reported, the conformational change of Fol-NCs toward the open state leads to a lower degree of stability^31^. Here we use Fol-scr-NCs as a model to initially study internalization and intracellular stability, since Fol-miR21-NCs, once entered in the cells, undergo a conformational change toward the bound open state due to the intracellular presence of miR-21 (Fig. 1).

**Fig. 2:**
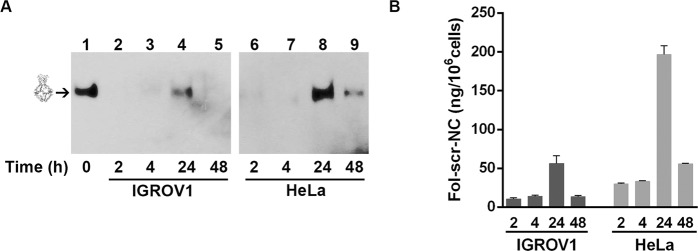
Time-dependent uptake of Fol-scr-NCs in HeLa and IGROV1 cells. **A** DNA blot of biotinylated-Fol-scr-NCs purified from IGROV1 (lanes 2–5) and HeLa (lanes 6–9) cell lysates at 2, 4, 24, and 48 h incubation time and detected with streptavidin-HRP. Lane 1 shows 30 ng of NCs before incubation with cells (time 0). **B** Densitometric analysis of the amount of Fol-scr-NCs internalized in cells at 37 °C at different times, as indicated, analyzed with the ImageJ software. Histograms show average values ± S.E.M. of three independent experiments.

Fig. 2A shows the DNA blot of nanocages internalized via αFR pathway. In detail, HeLa and IGROV1 cells were incubated with 6 μg/ml of biotinylated Fol-scr-NCs for different times. It is important to remark that we did not observe any difference in the internalization mechanism using a range of concentrations between 1.5 and 10 µg/ml of nanostructures. Fol-NCs were purified from cell lysates and analyzed by DNA blot using streptavidin-HRP^25^. Lane 1 of Fig. 2A shows the electrophoretic mobility of the input band of nanocages (30 ng) prior to the incubation with cells (time 0). A very low amount of Fol-scr-NCs is present after 2 and 4 h of incubation, while an evident band, corresponding to intact nanostructures, is detectable at 24 h in both IGROV1 and HeLa cells (Fig. 2A, lanes 4 and 8), confirming our previous observation that intact DNA nanocages accumulate inside the cells when uptaken by the folate-mediated pathway^27^. Of note, after 48 h, intact Fol-scr-NCs are still detectable inside HeLa cells (lane 9), while they are almost undetectable in IGROV1 cells (lane 5). The relative intensity of each DNA blot band was quantified by densitometric analysis, normalized to the intensity of the input and reported in Fig. 2B. The amount of internalized Fol-NCs after 24 h is 56.4 ± 9 ng/10^6^ cells in IGROV1 and 196.5 ± 11 ng/10^6^ cells in HeLa cells.

### Anti-miR-21 activity of Fol-miR21-NCs in folate receptor positive cancer cells

To prove whether Fol-miR21-NCs, once entered in cells, sequester miR-21 thus decreasing its free concentration and inhibiting its activity, qPCR analysis of miR-21 expression was performed using the ΔΔCt method and normalized to the levels of endogenous U6 snRNA. The analysis in untreated cells shows a 23 ± 6% higher expression level of miR-21 in HeLa compared to IGROV1 cells (Supplementary Fig. 3).

**Fig. 3:**
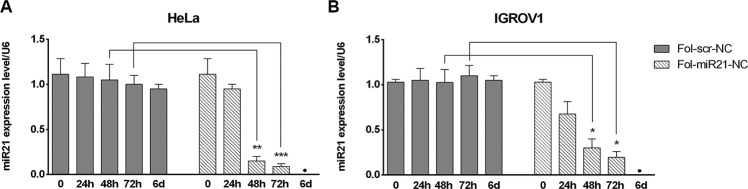
MiR-21-silencing activity in HeLa and IGROV1 cells of Fol-miR21-NCs compared to Fol-scr-NCs. qPCR analysis of miR-21 expression level is reported for HeLa (**A**) and IGROV1 (**B**) cells incubated with Fol-NCs for different time intervals. Values are expressed as mean ± S.E.M. of three different independent experiments. Statistical significance: ^*^P < 0.05, ^**^P < 0.01, and ^***^P < 0.001 (Student’s *t*-test). Values below detection limit (•).

HeLa and IGROV1 cells were incubated with 1.5 μg/ml of Fol-NCs, repeating the treatment every day since, according to serum stability data (Supplementary Fig. 2), the lifetime of Fol-NCs is about 16–18 h^31^. After 24 h treatment with Fol-miR21-NCs, a selective inhibition of miR-21 expression was observed in both HeLa and IGROV1 cells (15 ± 3% and 34 ± 9%, respectively). A much higher inhibition was observed at 48 h and 72 h, while, at longer time treatment (6 days), miR-21 level was not detectable. In detail, in HeLa cells the inhibition was 87 ± 4% at 48 h and 92 ± 3% at 72 h and in IGROV1 cells 70 ± 8% and 80 ± 6%, respectively. Treatment of cells with non-relevant Fol-scr-NCs, used as negative control, for the same time intervals did not affect miR-21 intracellular level (Fig. 3).

To compare the Fol-miR21-NC’s effects with an anti-miR-21 oligonucleotide, we transfected HeLa cells with anti-miR-21 or a scramble oligonucleotide and observed a maximum of 66 ± 6% decrease in miR-21 (Supplementary Fig. 5A).

### Therapeutic effects of Fol-miR21-NCs on cancer cells

**Fig. 4:**
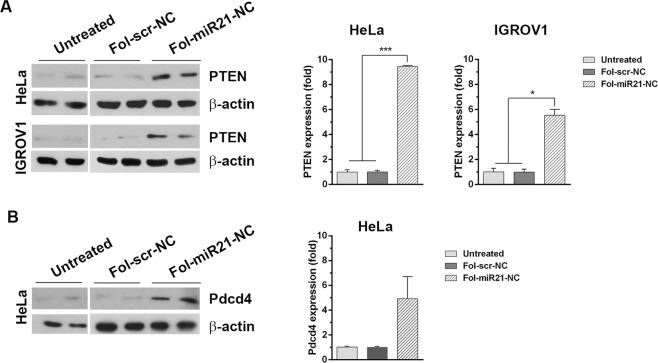
Expression level of PTEN and Pdcd4 miR-21 target proteins. **A** Western blots of PTEN in HeLa and IGROV1 cells lysates, treated or not with Fol-NCs for 48 h. **B** Western blot of Pdcd4 on lysates from HeLa cells treated or not with Fol-NCs for 72 h. Densitometric analysis of three different experiments are shown on the right panels. Values are expressed as mean ± S.E.M. Statistical significance: ^*^P < 0.05 and ^***^P < 0.001 (Student’s *t*-test).

To demonstrate the biological effects of miR-21 knockdown, we studied the expression level of PTEN and Pdcd4, two known downstream targets regulated by miR-21. HeLa and IGROV1 cells were incubated with 1.5 μg/ml Fol-miR21-NCs or Fol-scr-NCs, used as control, repeating the treatment every day. Figure 4A shows that the effect on PTEN expression by Fol-miR21-NCs is not evident after 24 h incubation, whereas a robust increase in PTEN level is observed after 48 h. Densitometric analysis shows that PTEN level is 9.5 ± 1-fold higher in Fol-miR21-NC treated HeLa cells and 5.5 ± 1-fold higher in Fol-miR21-NC treated IGROV1 cells versus negative controls.

Up-regulation of Pdcd4 is detectable only after 72 h incubation in HeLa cells. Densitometric analysis indicates that Pdcd4 expression level is 4.9 ± 2-fold higher in Fol-miR21-NC treated cells when compared to Fol-scr-NC treated cells (Fig. 4B, right panel). No variation in the expression level of Pdcd4 protein was observed in IGROV1 cells. One reason could be that alterations in Pdcd4 protein expression are reported to be associated with the development of chemoresistance in ovarian cancer cell lines^33^. It is worth noting that untreated and Fol-scr-NC treated cells show comparable expression levels of PTEN and Pdcd4 proteins (Fig. 4 and Supplementary Fig. 4).

As a comparison, by transfecting cells with an anti-miR-21 oligonucleotide for 24 h, we observe a transient 2.5-fold and 3-fold increase in PTEN level in HeLa and IGROV1 cells, respectively (Supplementary Figs. 5B and 6).

**Fig. 5:**
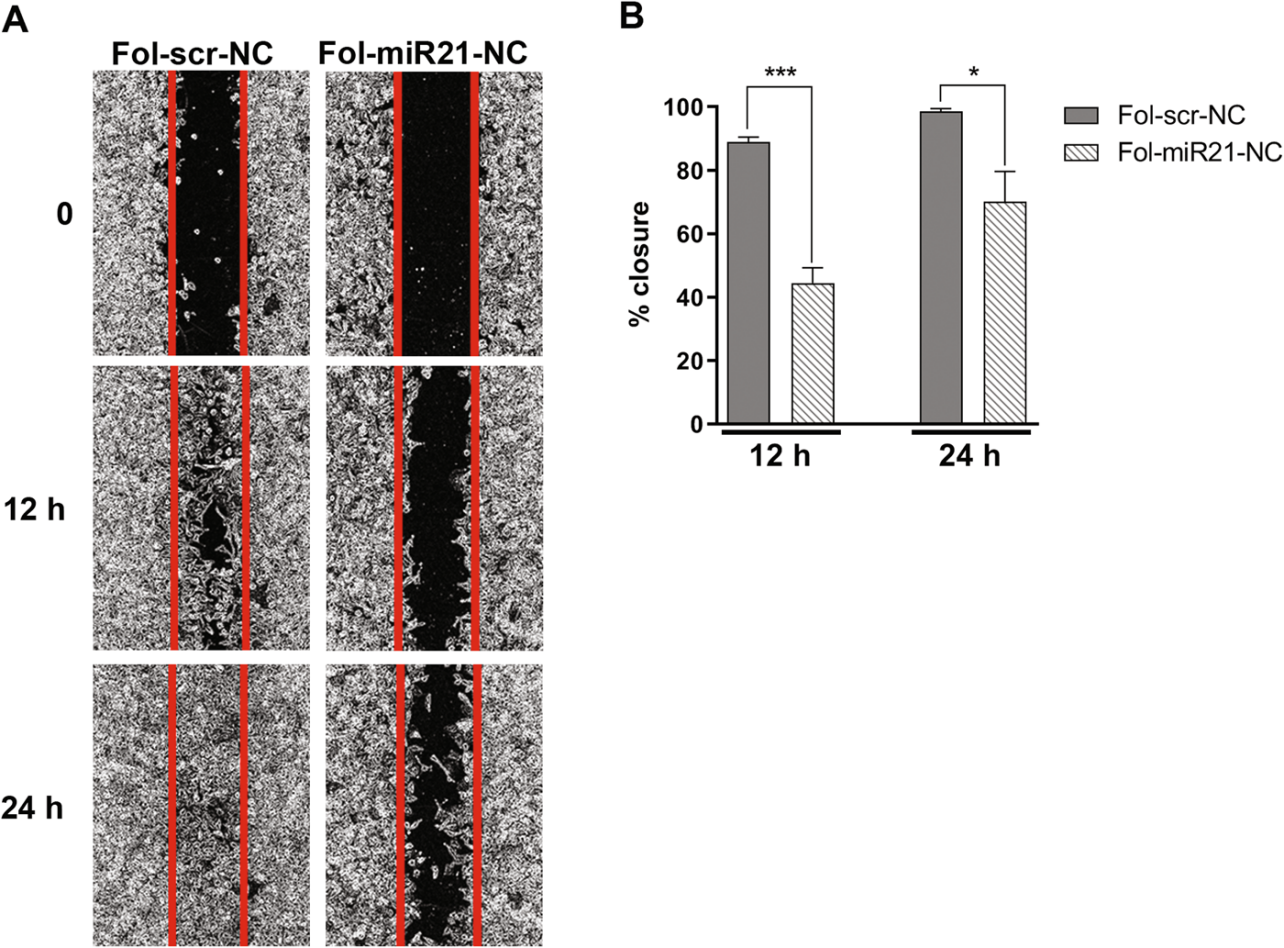
MiR-21 silencing inhibits migration of HeLa cells. **A** In vitro wound-healing assay on cell migration across the scratched area was monitored for 24 h. Images converted to grayscale are shown. **B** Histograms represent the relative percentage of distance covered by cells 12 and 24 h after scratch (% closure). Values reported were obtained as mean ± S.E.M. of three different experiments. Statistical significance: ^*^P < 0.05 and ^***^P < 0.001 (Student’s *t*-test).

The effects of miR-21 knockdown on cell migration has been evaluated through a wound-healing assay on HeLa cells. Cells were treated with 1.5 µg/ml of Fol-NCs for 48 h and, after creating a wound, the scratch width was measured by photo recordings at 0, 12, and 24 h. Fig. 5A shows that Fol-miR21-NC treated cells were less efficient in migrating and closing the space into a confluent cell monolayer compared to scrambled control NCs. Quantitative histograms in Fig. 5B show that, after 12 h, the closure of the scratched area by Fol-miR21-NC treated cells is 44 ± 5% compared to 89 ± 2% of Fol-scr-NC treated cells and, after 24 h, the closure is 70 ± 9% and 98.5 ± 1%, respectively.

**Fig. 6:**
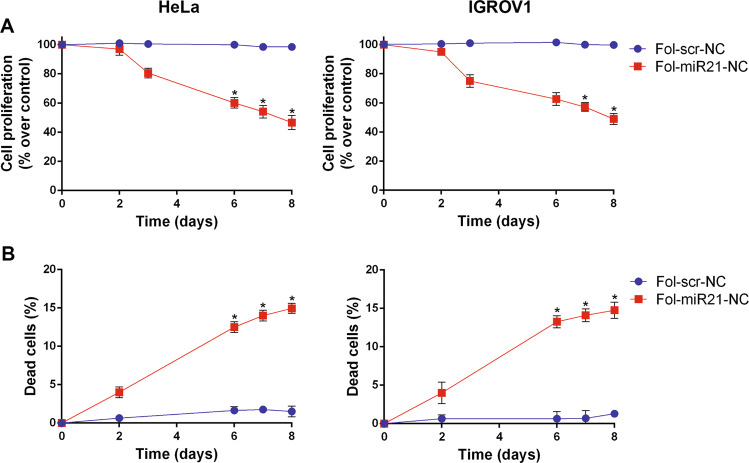
Cytotoxic effect of Fol-miR21-NCs in HeLa and IGROV1 cells. Cell proliferation after treatment with Fol-NCs at different times was assessed by MTS (**A**) and cell death was evaluated by trypan blue assay (**B**). The values are the mean of six replicates normalized to untreated cells. Statistical significance: ^*^P < 0.05 (Student’s *t*-test).

Anti-proliferative activity of Fol-miR21-DNA nanocages was tested by MTS assay, incubating HeLa and IGROV1 cells with 1.5 μg/ml nanocages for different times up to 8 days, repeating the treatment every day. Fig. 6A shows that the reduction in cell proliferation after 3 days was 20 ± 2% and 26 ± 3%, and reached 54 ± 3% and 51 ± 2% after 8 days of treatment in HeLa and IGROV1 cells, respectively. No effect was observed in cells treated with Fol-scr-NCs. In line with our previous observation^26^, Fol-miR21-DNA nanocages are selectively internalized in αFR-overexpressing cells and no reduction in cell proliferation was observed in cells that do not express the αFRs on their surface, such as human epidermoid carcinoma A431 or human embryonic kidney (HEK-293) cells (Supplementary Fig. 7). Notably, as expected, these cell lines do not internalize Fol-NCs (Supplementary Fig. 8).

On the other hand, in vitro cytotoxicity of Fol-miR21-NCs determined by trypan blue assay shows an increase in cell death from the first day of incubation, up to 15% after 8 days (Fig. 6B). Notably, both HeLa and IGROV1 cells respond similarly to the treatment, notwithstanding the fact that IGROV1 cells internalize less than one-third of DNA nanocages compared to HeLa cells (Fig. 2).

### Combined treatment with Fol-miR21-NCs and doxorubicin

**Fig. 7:**
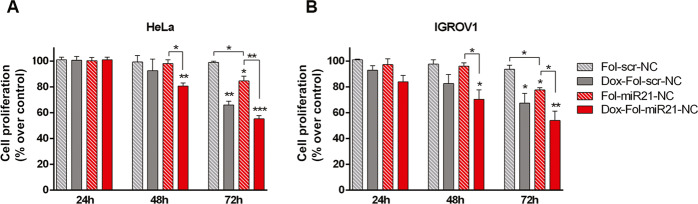
Effect of Fol-miR21-NCs and Dox combined treatment. Cell proliferation of HeLa (**A**) and IGROV1 (**B**) evaluated by MTS assay. Cells were treated with Dox-loaded and not-loaded Fol-miR21-NCs and Fol-scr-NCs at different times, as indicated. The data represent mean ± S.E.M. of three separate experiments. The values are the mean of six replicates, normalized on cell proliferation of untreated cells. Statistical significance: ^*^P < 0.05, ^**^P < 0.01, and ^***^P < 0.01 (Student’s *t*-test).

We finally explored the role of Fol-miR21-NCs as multipurpose structures for miRNA silencing and as drug carriers by loading nanocages with Dox^26^. By MTS assays we analyzed the cytotoxic effect of Fol-miR21-NCs, loaded or not loaded with Dox, in HeLa and IGROV1 cells (Fig. 7). Nanostructures were used at a basepair concentration of 4 μM. The estimated Dox concentration in Dox-loaded Fol-NCs was 0.44 ± 0.1 μM, considering that 22.5% of the initial Dox used for intercalation is present in NCs after purification (Supplementary Fig. 9). At 72 h incubation, Fol-miR21-NC treatment led to a cell proliferation reduction of 15 ± 4% and 22 ± 3% over the control (Fol-scr-NCs) in HeLa and IGROV1 cells, respectively. Notably, treatment with Dox-loaded Fol-miR21-NCs (Dox-Fol-miR21-NCs) induces an additive effect, which results in 45 ± 4% reduction in cell viability in HeLa and 46 ± 12% in IGROV1 cells. As expected, incubation of cells with control Fol-scr-NCs does not induce any toxic effect (see Fig. 6). Therefore, the observed toxicity of control-NCs loaded with doxorubicin (Dox-Fol-scr-NCs), corresponding to 35 ± 6% and 32 ± 9% of cell viability reduction in HeLa and IGROV1 cells, respectively, is due to the presence of Dox.

## Discussion

Here we demonstrate that Fol-NCs engineered with four sequestering units complementary to miR-21, for inhibiting miR-21 function, enter the cells through the αFR-mediated route and sequester miR-21 within cells, leading to 70–80% reduction in either IGROV1 or HeLa cells, after 48 h treatment. Considering that the amount of miR-21 present in HeLa cells is 4 × 10^3^ copies/10 pg total RNA^35^ corresponding to 0.12 ng/10^6^ cells and that the amount of internalized cages in HeLa is 200 ng/10^6^ cells (Fig. 2B), the molar ratio between Fol-miR21-NCs and endogenous miR-21 ranges between 60:1 and 30:1, depending on the experiment. Since each nanocage has four sequestering units, the “miR-21 sequestering units:endogenous miR-21” ratio ranges between 240:1 and 120:1, so there are always more than 100x molar excess of sequestering units. This ratio permits an efficient miR-21 binding, as recently demonstrated in vitro^31^.

Folic acid is a biocompatible and non-immunogenic compound internalized in the cytoplasm through αFR pathway for cellular utilization^36^. The advantage of using folate ligand compared to other targeting signals is that, when DNA nanostructures enter through the αFR pathway, they are stable for hours and slowly accumulate inside the cells without degradation^26,27^, likely due to the fact that folic acid is internalized for consumption rather than for destruction^36^. In line, Fig. 2 shows that the highest concentration of intact Fol-NCs inside the cells occurs after 24 h of incubation, unlike what happens to pristine H4-DNA-NCs administered by transfection^31^. In the latter case, most of the nanocages enter in 2–3 h and are then quickly degraded.

The Fol-miR21-NC’s anti-miR activity, measured by qPCR, is more evident after 48 h of treatment (>80% of inhibition) and miR-21 level remains very low for days, throughout the administration period of the nanostructures. Notably, the inhibition obtained with Fol-NCs is more efficient than that obtained with the anti-miR-21 oligonucleotide given by using a transfection reagent (>80% vs 66% reduction of miR-21 level over the control) and the anti-miR-21 oligonucleotide transfection has a faster but transient activity. Moreover, transfection agents are toxic^37^ and cannot be administered to cells continuously, whereas folate-functionalized nanocages are not cytotoxic per se^26^ and can be administered, added to the culture medium, daily for several days (Fig. 6).

Upon confirmation of efficient targeting, we studied the biological effects due to miR-21 silencing. MiR-21 is an overexpressed oncogenic gene in ovarian and cervix cancer and is associated with metastasis and poor prognosis^32,33^. In vitro studies have shown that down-regulation of miR-21 results in up-regulation of the tumor suppressor proteins PTEN and Pdcd4, and increased apoptosis^32,38^. In line with these findings, miR-21 knockdown by Fol-miR21-NCs leads to a marked up-regulation of PTEN and Pdcd4 proteins, respectively.

MiR-21 knockdown by Fol-miR21-NCs leads to inhibition of cell migration evaluated by measuring the scratched area’s closure, and to a reduction of cell viability in treated cells. It is interesting to note that both cell lines respond in a similar way to the cytotoxic action. The cell proliferation assays indicate a reduction in cell viability of 51% and 53% in HeLa and IGROV1, respectively. In line, similar effects have been obtained using other DNS as vehicles for the release of anti-miR-21 sequences or for direct oncomiRs-silencing activity^39–41^.

Our previous characterization of the intracellular localization of Fol-NCs by confocal analysis^26^ and of their intracellular traffic by colocalization with early endosomes and lysosome markers^27^, indicated that they enter through the folate receptor-mediated endocytosis pathway, allowing their accumulation into the cytoplasm, never going into the nucleus and so excluding that the structures interfere with pri- and pre-miR21 levels.

Finally, Fol-miR21-NCs are an efficient binding platform for intercalating drugs, such as Dox. The loading procedure is easy and the stability of Dox-loaded NCs at physiological pH indicates to the potential application of DNA nanocages as drug nanocarriers^26^. Folate receptor-mediated entry of Dox-loaded control nanocages (Fol-scr-NCs) results in elevated cytotoxicity, confirming our previous data^26^. Here we show that combination of miR-21 sequestering and Dox delivery enhances the cytotoxicity of Fol-miR21-NCs, in an additive way.

In conclusion, the here described self-assembled Fol-miR21-NCs, used as delivery vehicle for sequestering intracellular oncogenic miR-21 are very stable and present several advantages. The four oligonucleotides containing miRNA sequestering units can be modified with DNA sequences complementary to different oncomiRs to enlarge the possible applications, and DNA-NCs can be functionalized with different ligands for a selective non-toxic targeting, paving the way for their use as selective nucleic acid drugs. Resistance of cancer cells to therapy along with the occurrence of severe side effects of commonly used treatments have raised the urgency in the development of new and safe anti-cancer agents. Currently, treatments that combine chemotherapeutic drug delivery with gene silencing are emerging as a promising therapeutic option. Multifunctional DNA-based nanocages, here described, engineered to simultaneously achieve selective cell targeting, specific oncomiRs sequestering and drug delivery, can be one of the eligible systems to be applied in cancer therapy to overcome major challenges.

## Materials and methods

### Preparation of functionalized octahedral DNA nanocages

Folate-functionalized nanocages harboring miR-21 sequestering units (Fol-miR21-NCs) or scramble sequences (Fol-scr-NCs) were assembled and purified as described earlier^26,31^. A biotin molecule was added on one edge of the structure for the detection of NCs through the streptavidin (HRP)−biotin reaction. Oligonucleotide sequences used for the assembly of NCs are reported in Supplementary Information 10 and Supplementary Table 1.

### Cell cultures

HeLa cells (provided by M. Figini, Istituto Nazionale Tumori, Milano, Italy) derived from human cervix cancer, were grown in DMEM (Dulbecco’s modified Eagle’s medium) (Biowest, Miami, FL, USA) and IGROV1 cells (provided by M. Figini, Istituto Nazionale Tumori, Milano, Italy) derived from ovarian carcinoma in RPMI 1640 (Euroclone, Devon, UK), supplemented with 10% FBS (Gibco, Paisleg, UK), 1 mM L-glutamine (Sigma Aldrich, St Louis, MO, USA), 1 mM sodium pyruvate (Biowest, Miami, FL, USA), and 100 U/ml penicillin-streptomycin (Euroclone, Devon, UK). Cells were authenticated by STR profiling and periodically tested for mycoplasma contamination.

### Purification of DNA nanocages from cell lysates and DNA blot

Cells were plated in 48-well plates at a density of 3 × 10^4^ cells/well and grown in folate-free RPMI 1640 (Sigma Aldrich, St Louis, MO, USA) supplemented with 10% FBS for 24 h. Experiments were performed in folate-free RPMI 1640 medium with 2% FBS. Cells were lysed, centrifuged, digested with proteinase K, and analyzed by DNA blot, as described earlier^25^. Detection of biotinylated-NCs was carried out using streptavidin-HRP (Horseradish Peroxidase) (Abcam Inc., Toronto, ON, Canada, Ab7403) and visualized by enhanced chemiluminescence (ECL Extend, Euroclone, Devon, UK). Input samples of NCs are DNA structures added to cell culture medium and immediately digested with proteinase K and processed for DNA blot.

### RNA isolation and qPCR for miR-21 expression analysis

Total RNA was extracted using RNeasy Mini Kit (Qiagen, Hilden, Germany) and reverse transcribed into cDNA by using miScript II RT Kit (Qiagen, Hilden, Germany). RNA concentration was determined using NanoDrop spectrophotometer (NanoDrop ND-1000 Waltham, Massachusetts, MA, USA). For quantitative analysis of miR-21 expression, qPCR amplification of cDNA was performed using miScript Primer assay and miScript SYBR Green PCR Kit (Qiagen, Hilden, Germany) on a Real-Time PCR Detection System (Bio-Rad, Hercules, California,CA, USA), following the manufacturer’s instruction. Relative transcript quantification of miR-21 was determined using the ΔΔCt method, normalized to the levels of endogenous U6 snRNA and to the untreated control cells.

### Western blot

Cells were lysed and centrifuged as described previously^31^. The supernatant fraction was analyzed by SDS-polyacrylamide gel electrophoresis in 10% acrylamide gels and blotted (Trans Blot Turbo Bio-Rad Laboratories). PTEN rabbit mAb (Cell Signaling Technologies, Danvers, MA, USA, cat. n. 138G6), Pdcd-4 mouse mAb (Santa Cruz Biotechnology, Dallas, Texas, USA, cat. n. SC-376430), and β-actin mouse mAb (Cell Signaling Technologies, Danvers, MA, USA, cat. n. 8H10D10) were used as primary antibodies. HRP-conjugated AffiniPure goat anti-mouse IgG (Jackson Immunoresearch, Cambridgeshire, UK, cat. n. 115-035-062) and HRP-conjugated AffiniPure donkey anti-rabbit IgG secondary antibody (Jackson Immunoresearch, Cambridgeshire, UK, cat. n. 711-035-152) were used as secondary antibodies. Immunoreactive bands were visualized by enhanced chemiluminescence (ECL Extend, Euroclone, Devon, UK). The ECL data were scanned and analyzed by ImageJ software. Band intensities were normalized to β-actin.

### Doxorubicin intercalation

Fol-NCs’ intercalation with Dox (Enzo Life Sciences Farmingdale, NY, USA) and purification procedure were performed as described previously^26^. The amount of intercalated Dox was calculated as described in Supplementary Information 9.

### Cell viability assays

HeLa and IGROV1 cells were plated in 96-well plates at a density of 2 × 10^3^ cells/well, incubated with folate-free RPMI 1640 with 10% FBS for 24 h and treated with Fol-NCs. Treatment was repeated every 24 h. For longer time points, cells were treated as described above and, after 3 days, detached from wells by trypsinization and plated on 96-well plates. Cells were further treated with Fol-NCs for up to 8 days.

Cell proliferation was evaluated by using the 3-(4,5-dimethylthiazol-2-yl)-5-(3-carboxymethoxyphenyl)-2-(4-sulfophenyl)-2H-tetrazolium (MTS) assay (Promega, WI, USA). Absorbance was measured at 492 nm using Multiskan Ascent 96/384 Plate Reader (MTX Lab Systems, Bradenton, FL, USA). Trypan blue staining assay (Sigma Aldrich, St Louis, MO, USA) was performed by using a cell counting chamber.

### In vitro migration assay

HeLa cells were seeded at a density of 1 × 10^4^ cells/well in ibidi Culture-Insert 2 Well (ibidi GmbH, Gräfelfing, Germany) and grown in folate-free RPMI 1640 with 10% FBS overnight. Cells were incubated with Fol-NCs in folate-free RPMI 1640 with 2% FBS for 48 h. After the wound was created, the scratch width was measured by photo recordings at 0, 12, and 24 h using IncuCyte^®^ S3 Live-Cell Analysis System (Sartorius, Gottinga, Germany) and images captured using a 4x magnification objective and processed using ImageJ software. Images were converted to grayscale (Image-Type-8bit) and edges enhanced to highlight sharp changes in intensity (Process-Find Edges).

### Statistical analysis

Each experimental point was carried out in duplicate and repeated at least in three independent experiments (*n* ≥ 6). Data were analyzed using GraphPad Prism. Results are expressed as a mean ± S.E.M. and statistical analyses performed using Student’s *t*-test. Differences were considered statistically significant when *P < 0.05, **P < 0.01, and ***P < 0.001.

## Supplementary information

Supplementary Figure Legends

sFig.1

sFig.2

sFig.3

sFig.4

sFig.5

sFig.6

sFig.7

sFig.8

sFig.9

sTable 1
