# Supplementary material for: Combined and selective miR-21 silencing and doxorubicin delivery in cancer cells using tailored DNA nanostructures
Source: Cell Death Dis. 2021 Jan 7;12(1):7. doi: 10.1038/s41419-020-03339-3 (PMC7791072; doi:10.1038/s41419-020-03339-3)
Supplement: Supplementary file 1 — Supplementary Figure Legends [file 41419_2020_3339_MOESM1_ESM.docx]

**Supplementary figure legends**

**Figure 1.** A) Schematic representation of a truncated octahedral nanocage with four miR21 sequestering units, in which each oligonucleotide is visualized with a different color. B) Gel electrophoretic result of a ladder experiment where an increasing number of DNA oligonucleotides that form the cages have been annealed before analysis. Lanes 1−7 show the result of subjecting assembly reactions containing OL1−2, 1−3, 1−4, 1−5, 1−6, 1−7, and 1−8 oligonucleotides and lane M contains the DNA marker. Arrow shows the band in lane 7, corresponding to the assembled nanostructure.

**Figure 2.** DNA blot analysis. 30 ng of nanocages before incubation with serum proteins (time 0) are shown in each panel. Biotinylated nanocages are detected by using Streptavidin-HRP.

**Figure 3.** qPCR analysis of miR21 endogenous levels in HeLa and IGROV1 cell lines. Relative transcript quantification of miR21 was determined using the ΔΔCt method, normalized to level of endogenous U6 snRNA. Histograms show means ± S.E.M. of three different experiments.

**Figure 4.**  Western blot analysis of PTEN protein level in HeLa (A) and IGROV1 (B) cells incubated without or with Fol-scr-NCs for 48 h. (C) Western blot analysis of Pdcd4 protein expression level in HeLa treated without or with Fol-scr-NCs for 72 h. β-actin was used as internal control.

**Figure 5.** Transfection of anti-miR21 oligonucleotide in HeLa cells A) qPCR showing effect of anti-miR21 transfection on the endogenous miR21 levels. B) Western blot of PTEN protein. β-actin was used as internal control. Densitometric analysis is shown in the left panel, histograms represent the average ± S.E.M. of three experiments, Statistical significance: P < 0.05 (*) (Student’s t-test).

**Figure 6.** Western blotting of PTEN on IGROV1 cell lysates. β-actin was used as internal control. Densitometric analysis is shown in the left panel, histograms represent the average ± S.E.M. of three experiments.

**Figure 7.** MTS assay of (A) A431 and (B) HEK-293 cells treated with Fol-NCs for 24, 48 and 72 h.

**Figure 8.** DNA Blot of biotinylated Fol-scr-NCs purified from HeLa (lane 2), A431 (lane 3) and HEK-293 (lane 4) cell lysates. Biotinylated Fol-scr-NCs were detected with streptavidin-HRP.

**Figure 9.** Standard curve of free Dox.

**Supplementary table legends**

**Table 1.** Sequences of the oligonucleotides used for the assembly of biotinylated Fol-scr-NCs and Fol-miR21-NCs. In blue are shown the two T in OL8 used for folate conjugation and in red is depicted the biotinylated T in OL6_BIO_.
