# Supplementary figures and images for: Combined and selective miR-21 silencing and doxorubicin delivery in cancer cells using tailored DNA nanostructures
Source: Cell Death Dis. 2021 Jan 7;12(1):7. doi: 10.1038/s41419-020-03339-3 (PMC7791072; doi:10.1038/s41419-020-03339-3)

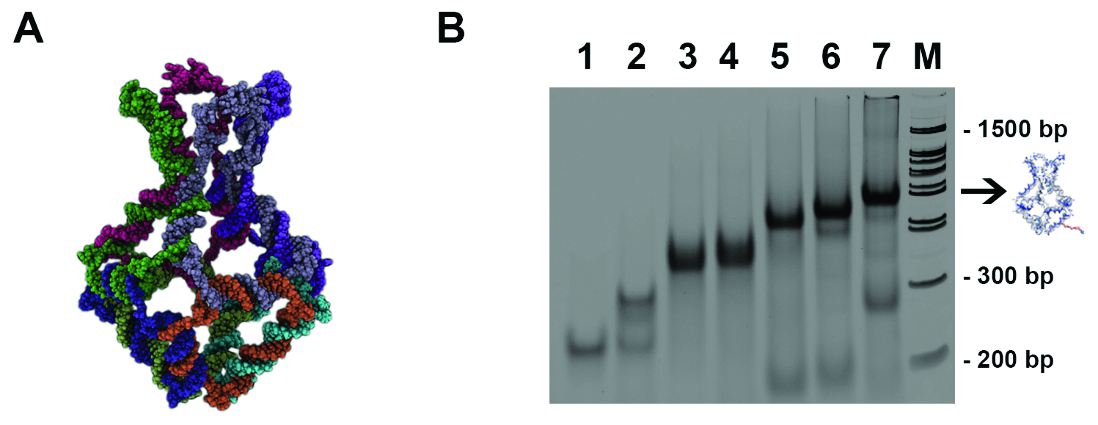

Supplement: Supplementary file 2 — sFig.1 [file 41419_2020_3339_MOESM2_ESM.tif]

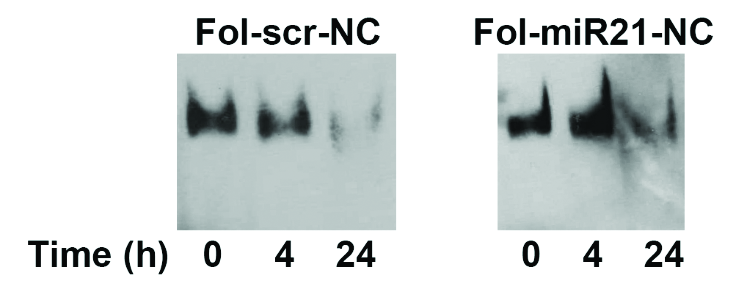

Supplement: Supplementary file 3 — sFig.2 [file 41419_2020_3339_MOESM3_ESM.tif]

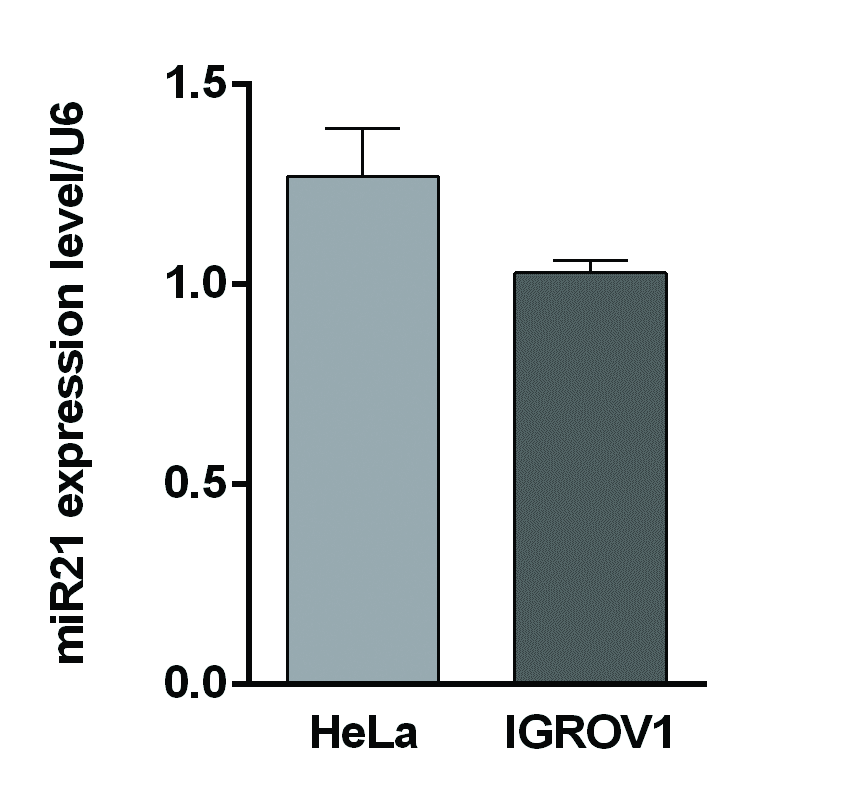

Supplement: Supplementary file 4 — sFig.3 [file 41419_2020_3339_MOESM4_ESM.tif]

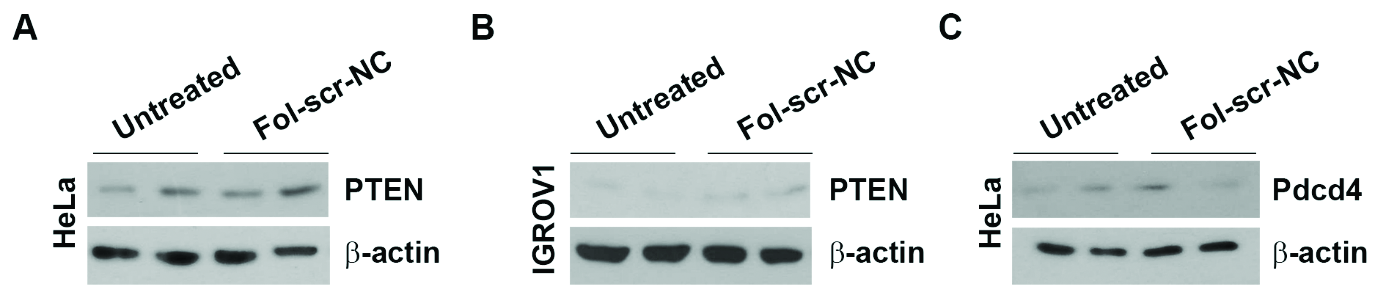

Supplement: Supplementary file 5 — sFig.4 [file 41419_2020_3339_MOESM5_ESM.tif]

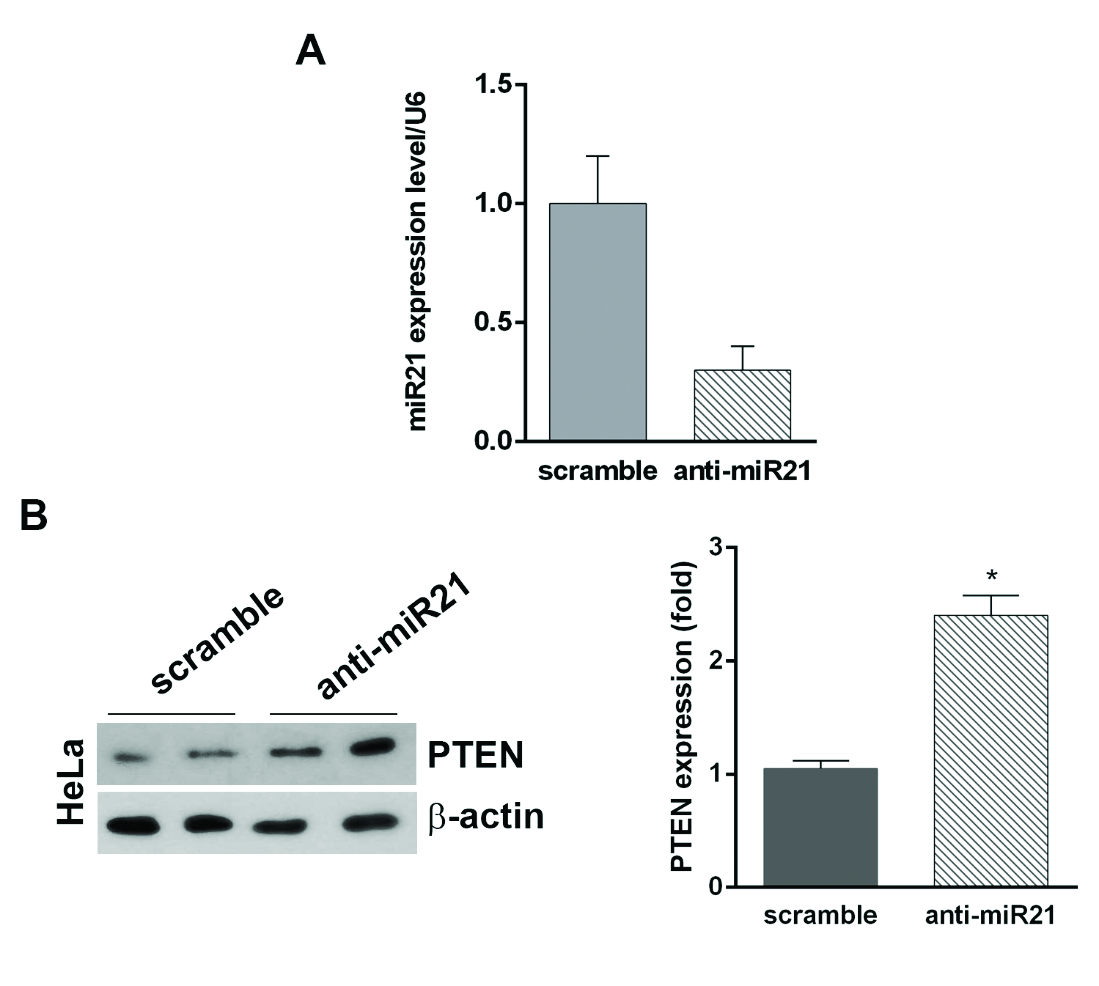

Supplement: Supplementary file 6 — sFig.5 [file 41419_2020_3339_MOESM6_ESM.tif]

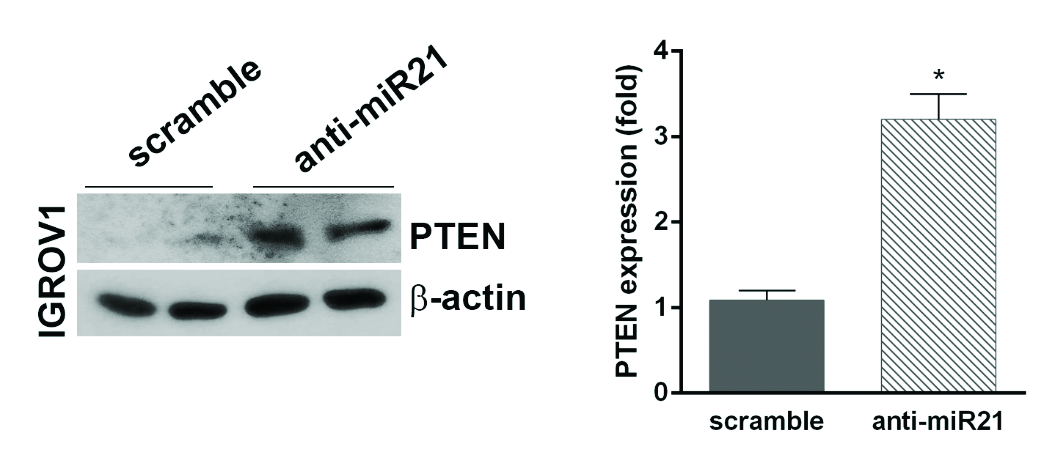

Supplement: Supplementary file 7 — sFig.6 [file 41419_2020_3339_MOESM7_ESM.tif]

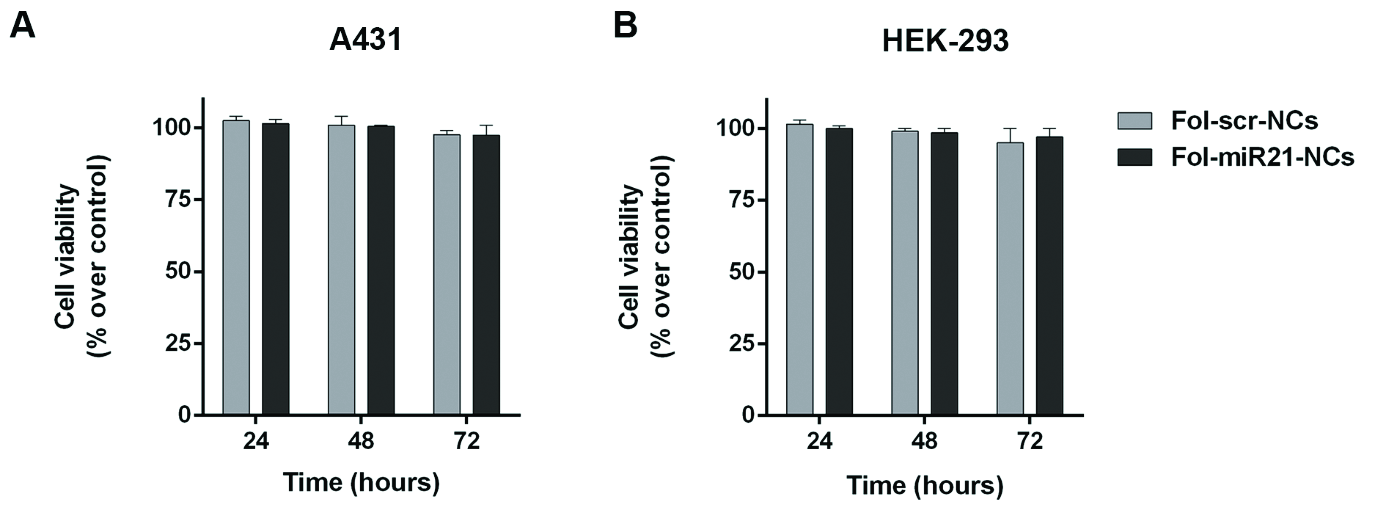

Supplement: Supplementary file 8 — sFig.7 [file 41419_2020_3339_MOESM8_ESM.tif]

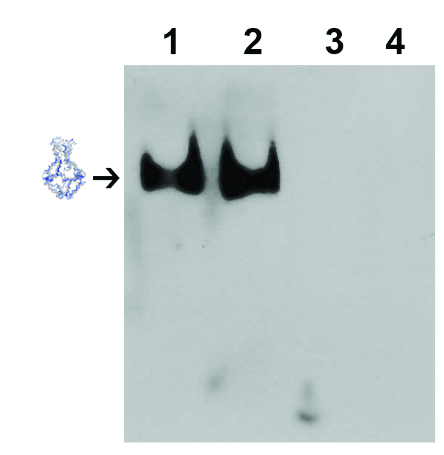

Supplement: Supplementary file 9 — sFig.8 [file 41419_2020_3339_MOESM9_ESM.tif]

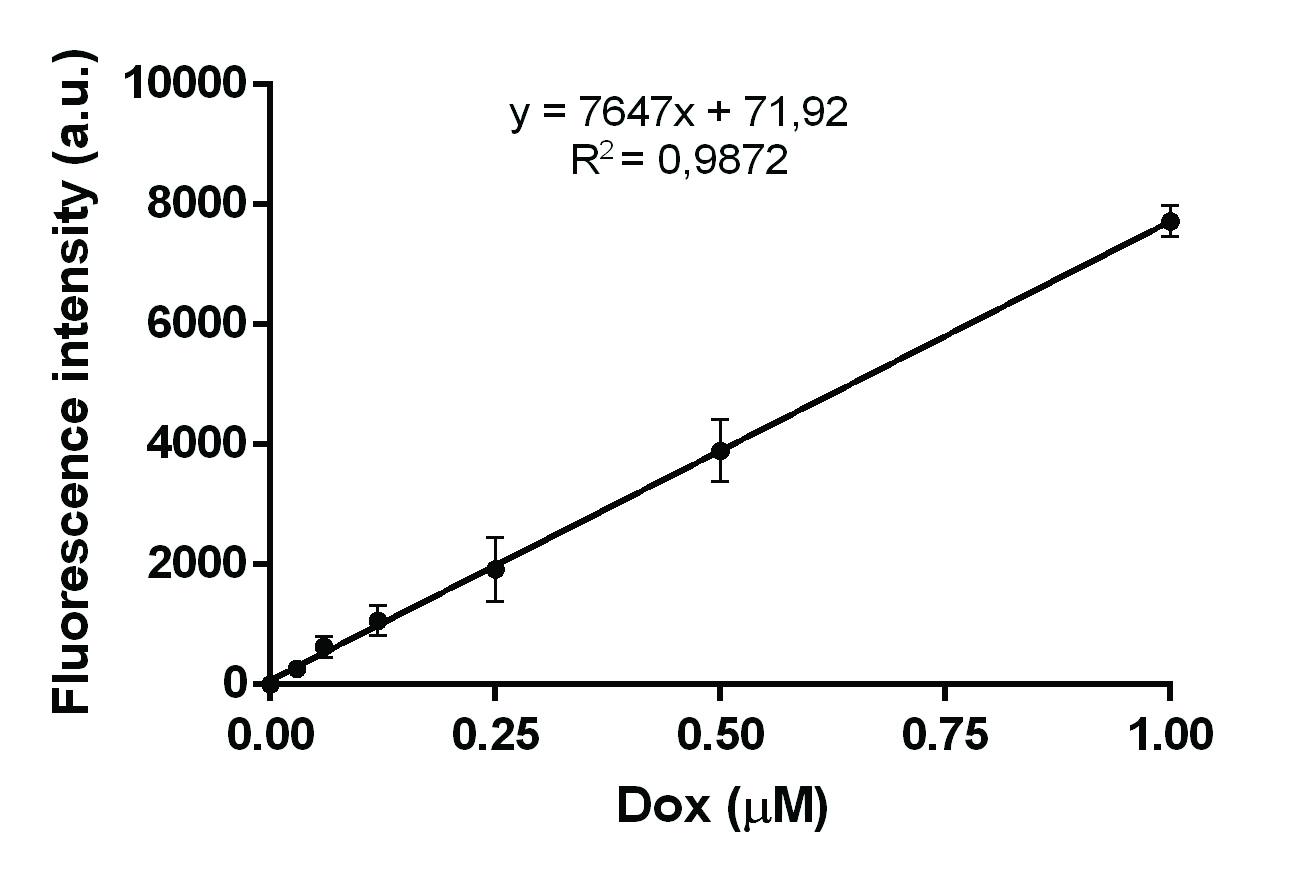

Supplement: Supplementary file 10 — sFig.9 [file 41419_2020_3339_MOESM10_ESM.tif]
