# Supplementary material for: Combined and selective miR-21 silencing and doxorubicin delivery in cancer cells using tailored DNA nanostructures
Source: Cell Death Dis. 2021 Jan 7;12(1):7. doi: 10.1038/s41419-020-03339-3 (PMC7791072; doi:10.1038/s41419-020-03339-3)
Supplement: Supplementary file 11 — sTable 1 [file 41419_2020_3339_MOESM11_ESM.docx]

| **Oligo** | **Sequences (5’-3’)** |
| --- | --- |
| OL1_miR_ | GCCACCAGGTTTTTCGATGTCTAAGCTGACCGTCTTTCCTTTTTCAACATCAGTCTGATAAGCTATTTCCTTTTCTGGACCGTGATTCCATGACTTTTTCTTAGAGTT |
| OL2_miR_ | TGGCTACAGTCTTTCCTTTTTCAACATCAGTCTGATAAGCTATTTCCTTTTCTCGGTCAGCTTAGACATCGTTTTTGAATCCTATGCTCGGACGTTTTTGGCTCACAT |
| OL3_miR_ | TCACGGTCCTCTTTCCTTTTTCAACATCAGTCTGATAAGCTATTTCCTTTTCTCTATCCGATCGAGGCATGTTTTTCATACTGAGAGCGTTCCGTTTTTGTCATGGAA |
| OL4_miR_ | CAGATACGCTTTTTCATGCCTCGATCGGATAGTCTTTCCTTTTTCAACATCAGTCTGATAAGCTATTTCCTTTTCTCTGTAGCCAATGTGAGCCTTTTTGTCGCAGTT |
| OL5 | CTCAGTATGTTTTTCGGTTACGGTACAATGCCTTTTTGCCAAGACGTTAGTGTCCTTTTTCGGAACGCT |
| OL6_BIO_ | GGTGTATCGTTTTTGGCATTG**T**ACCGTAACCGTTTTTGCGTATCTGAACTGCGACTTTTTCCACCGAAT |
| OL7 | CGTCTTGCGTTTTTGTATGACGCAGCACTTGCTTTTTCCTGGTGGCAACTCTAAGTTTTTGGACACTAA |
| OL8_FOL_ | ATAGGATTCTTTTTGCAAGTGC**T**GCGTCATACTT**T**TTCGATACACCATTCGGTGGTTTTTCGTCCGAGC’ |
| anti-miR21 | MeOU-MeOC-MeOA-MeOA-MeOC-MeOA-MeOU-MeOC-MeOA-MeOG-MeOU-MeOC-MeOU-MeOG-MeOA-MeOU-MeOA-MeOA-MeOG-MeOC-MeOU-MeOA |
| OL1_scr_ | GCCACCAGGTTTTTCGATGTCTAAGCTGACCGTCGAATATTTCCCCCCCCCCAGAAACCTTCCTGGACCGTGATTCCATGACTTTTTCTTAGAGTT |
| OL2_scr_ | TGGCTACAGTCGAATATTTCCCCCCCCCCAGAAACCTTCCTCGGTCAGCTTAGACATCGTTTTTGAACTCTATGCTCGGACGTTTTTGGCTCACAT |
| OL3_scr_ | TCACGGTCCAATATTTCCCCCCCCCCAGAAACCTTCCTCTATCCGATCGAGGCATGTTTTTCATACTGAGAGCGTTCCGTTTTTGTCATGGAA |
| OL4_scr_ | CAGATACGCTTTTTCATGCCTCGATCGGATAGTCGAATATTTCCCCCCCCCCAGAAACCTTCCTCTGTAGCCAATGTGAGCCTTTTTGTCGCAGTT |

**Table 1.**
